# Deep learning for mango leaf disease identification: A vision transformer perspective

**DOI:** 10.1016/j.heliyon.2024.e36361

**Published:** 2024-08-22

**Authors:** Md. Arban Hossain, Saadman Sakib, Hasan Muhammad Abdullah, Shifat E. Arman

**Affiliations:** a Department of Robotics and Mechatronics Engineering, University of Dhaka, Dhaka 1000, Bangladesh; b GIS and Remote Sensing Lab, Department of Agroforestry and Environment, Bangabandhu Sheikh Mujibur Rahman Agricultural University, Gazipur 1706, Bangladesh

**Keywords:** Vision transformer, Plant disease, Deep learning, Smart agriculture

## Abstract

Over the last decade, the use of machine learning in smart agriculture has surged in popularity. Deep learning, particularly Convolutional Neural Networks (CNNs), has been useful in identifying diseases in plants at an early stage. Recently, Vision Transformers (ViTs) have proven to be effective in image classification tasks. These architectures often outperform most state-of-the-art CNN models. However, the adoption of vision transformers in agriculture is still in its infancy. In this paper, we evaluated the performance of vision transformers in identification of mango leaf diseases and compare them with popular CNNs. We proposed an optimized model based on a pretrained Data-efficient Image Transformer (DeiT) architecture that achieves 99.75% accuracy, better than many popular CNNs including SqueezeNet, ShuffleNet, EfficientNet, DenseNet121, and MobileNet. We also demonstrated that vision transformers can have a shorter training time than CNNs, as they require fewer epochs to achieve optimal results. We also proposed a mobile app that uses the model as a backend to identify mango leaf diseases in real-time.

## 1 Introduction

Mango (*Mangifera indica*) is a tropical fruit native to the Indian subcontinent, southern Asia and the Andaman Islands. Its origin dates back to 500 B.C. [27]. Mango is the national fruit of India, Pakistan and Philippines. In a significant part of the world, it has been titled the “King of Fruits”. Mangoes, along with mangosteens and guavas, rank sixth as the most produced fruit in the world [9]. As reported by FAO in 2020 [9], India accounts for 24 million tonnes of mango production, the most in the world.

Mango fruit contains both macronutrients and micronutrients. They have been proved to be a rich source of vitamins, minerals, and antioxidants. It is a good source of dietary fiber and contains a high amount of polyphenols [38]. Reports [15] have shown that mangoes possess anti-cancer and anti-diabetic properties. The fruit also has a positive effect on the skin and hair.

Mangoes, like any other crop, are susceptible to various diseases that can affect their growth, fruit quality, and overall yield. Mango cultivars differ with climate, geographical location and soil type. Over several hundred types of mango are known, with India cultivating over 1000 varieties [18]. Successful cultivation of one variety in a region does not guarantee the same for another. Hence, steps taken to control diseases and pests also vary from region to region. The nature, frequency, and severity of these diseases depend on the environment, mostly, and also the production process. These diseases impact the fruit yield, quality, and production cost of the tree.

Most mango diseases are caused by fungi and bacteria. Anthracnose, caused by the fungus *Colletotrichum gloeosporioides*, affects most tropical fruits. It is responsible for the most amount of post-harvest losses worldwide [3]. Other fungal diseases include, but are not limited to - dieback (caused by the pathogen *Botryosphaeria disrupta*), black rot (caused by *Ceratocystis paradoxa*), gall (*Fusarium decemcellare*), sooty mold (*Capnodium mangiferae*). Most prevalent bacterial diseases include bacterial canker (caused by *Xanthomonas campestris*), bacterial rot (*Pectobacterium carotovorum*), and crown gall (*Agrobacterium tumefaciens*) among many others [32].

Farmers have used visual inspection and chemical controls in the past to detect and prevent these diseases. However, these methods are time-consuming and prone to errors. With advancements in smart agriculture, usage of methods such as digital image processing and machine learning have become more common. These methods are more accurate, faster and less expensive to implement.

**Figure 1:**
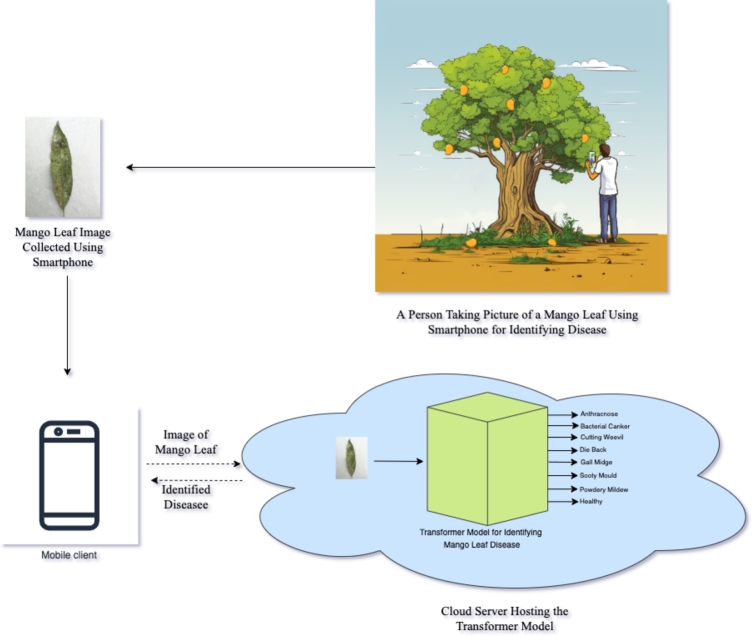
Automated Identification of Mango Leaf Disease Using Transformer Model.

In this work, we proposed using vision transformer models to identify seven different mango leaf diseases. We also optimized one vision transformer model to show that it can have a smaller size without sacrificing accuracy. A visual summary of our proposed work is presented in Fig. 1. The main contributions of this study are as follows:

1. We presented a fast and optimized vision transformer architecture to diagnose seven prominent mango leaf diseases. The model achieves a state-of-the-art accuracy of 99.75% on the MangoLeafBD [1] dataset.
2. We presented an extensive comparison between several Convolutional Neural Network (CNN) and Vision Transformer architectures for mango leaf diseases identification.
3. We developed a mobile app that uses the Optimized DeiT model as a backend to identify mango leaf diseases in real-time. This makes the model accessible to farmers and researchers.

The paper is further divided into 5 sections. In Section 2, we review the relevant literature. Section 3 first describes the dataset used for the study and the preprocession steps. We then discuss the proposed methodology, where we optimize several pre-trained CNN and transformer architecture to identify mango leaf diseases. In Section 4, we present the results and compare the networks. Section 5, discusses those result to provide insight. The paper concludes in Section 5.

## 2 Related work

### 2.1 Leaf disease recognition using machine learning

For a significant portion of the last decade, identification of leaf diseases has relied upon common machine learning techniques. Merchant et al. [23] developed an unsupervised machine learning model using K-means clustering to detect nutrient deficiencies in mango leaves. The model relies upon the color of the leaves. Most nutrient deficiency alter the natural green color of the leaf. A dataset is prepared after extracting the RGB values of the images. The model then classifies them into four groups (knows as clusters). Aslam et al. [6] proposed a similar solution for identifying varieties of healthy mango leaves. They used a KNN classifier with k=10. It achieved an average accuracy of 93.875% across 8 classes of mango leaves. Mia et al. [24] used a combination of Artifical Neural Networks (ANN) and Support Vector Machines (SVM) to extract interested regions of diseased leaves from the color space of their images. After separating images in groups using a K-means clustering algorithm, 13 features were extracted from these regions. This data is then passed to an SVM. Using this method, they achieved an average accuracy of 80%, being able to differentiate between four different types of disease.

### 2.2 Leaf disease recognition using deep learning

Arivazhagan et al. [4] used a Convolutional Neural Network (CNN) to identify five different leaf diseases. CNNs are designed to work with pixel data and extensively used in image recognition. They consist of multiple connected layers. Each of them build on top of previous layers and learned features. The CNN in [4] achieved an accuracy of 96.67%, claiming to be effective in real-time applications. Further improvement is possible using transfer learning, as shown by Arya et al. [5]. They used AlexNet, a pre-trained CNN architecture, and compared its performance against a regular CNN. They found the regular CNN to be only 90.85% accurate over a dataset of 4004 images of mango and potato. On the contrary, AlexNet achieved an accuracy of 98.33%. Mohanty et al. [26] also showed similar improvement gained through using transfer learning on AlexNet and GoogLeNet architectures. Over a dataset of 54,306 images, models trained from scratch were 96.72% on average, whereas models using transfer learning achieved 98.67% accuracy on average. Moreover, the GoogLeNet architecture was more effective than the AlexNet architecture as it consistently achieves an accuracy of 99%. Bhuiyan et al. [7] used Bayesian optimization to fine tune several pre-trained CNN models for diagnosing diseases from banana leaf images. They succeeded in developing a lightweight and deployable model that achieves 95.13% accuracy over three different disease classes. Prabu et al. [31] used another pre-trained model, MobileNetV2, with a crossover-based levy flight algorithm for feature selection. MobileNetV2 performs well on mobile devices, allowing lightweight deployments [35]. The model achieves an accuracy of 94.5%, identifying 3 different mango leaf diseases. Rizvee et al. [33] use a deep CNN architecture built on top of AlexNet which achieves 99% accuracy on the MangoLeafBD dataset. They optimize the existing AlexNet architecture to contain a lesser number of channels and be lightweight. Mahmud et al. [21] also follow a similar pattern by training a pretrained DenseNet architecture to predict images from the MangoLeafBD dataset. Their model also achieves a high accuracy of 99% over the test set.

### 2.3 Vision transformers

Vision Transformers (ViT) apply the logic of the regular Transformer architecture in image classification tasks. Images are reshaped into a sequence of flattened 2D patches, which are then used as input for a pure Transformer. Dosovitskiy et al. [8] claim in their proposal that ViT achieves greater results than state-of-the-art pre-trained CNN architectures while using fewer computational resources. In the recent years, using ViT in plant disease classification problems have been experimented. Thai et al. [39] discuss a potential superiority of ViT over established methods in analyzing cassava leaf diseases. They build a system relying on ViT that achieves F1-scores ranging from 75 to 96 over five categories. They claim that this model is at least 1% more accurate than popular CNN models, e.g. EfficientNet or ResNet. Alshammari et al. [2] push this idea to identify olive diseases in. They compare a simple ViT and a CNN, and show that the ViT model obtains 95% accuracy in detecting five diseases of olive. Thakur et al. [40] used a combination of ViT and CNN to classify diseases among a wide range of plants. Their hybrid model achieves 98.61% accuracy on the PlantVillage dataset and 87.87% accuracy on the Embrapa dataset.

## 3 Methodology

### 3.1 Dataset description

4,000 mango leaf images were sourced from the MangoLeafBD [1] dataset. These images are categorized into eight classes: seven for different diseases and one for healthy leaves.

Anthracnose causes black patches along the leaf margin and curl along the edges [3]. Bacterial Canker produces watery spots on almost all parts of a mango tree [25] including fruit, leaves, and even branches. Cutting Weevil makes the leaves look like they are cut cleanly with scissors [10]. Dieback causes the leaves to turn yellow [16] and fall off. Gall Midge introduces pimple-like spots on leaves [1]. Powdery Mildew, unlike others, is a fungal infection [29] on the leaf's surface. Sooty mould grows on the honeydew secreted by insects, slowly spreading over the entire leaf and causing it to turn to black [1]. Healthy leaves are free from any of these diseases.

Each class contains 500 images. The dataset was split into three sets. 80% of the images were used for training, 10% for validation and 10% for testing (Table 1). The splitting was done randomly.

**Table 1:** Distribution of train, test, and validation set.

Class	Train	Validation	Test
Anthracnose	400	50	50
Bacterial canker	400	50	50
Cutting weevil	400	50	50
Die back	400	50	50
Gall midge	400	50	50
Powdery Mildew	400	50	50
Sooty Mould	400	50	50
Healthy	400	50	50

### 3.2 Deep learning models

We used five pretrained CNNs and two pretrained ViTs to perform the image classification task. The CNN models are lightweight as they are to be deployed in mobile devices. Transformers usually have larger size compared to their CNN counterparts [28]. To solve this, we further optimized the pretrained ViT model to have a smaller size, while maintaining a similar accuracy. Details of the optimization are discussed in 3.5.

#### 3.2.1 CNN models

SqueezeNet [13] is a deep neural network (DNN) architecture that takes AlexNet as a reference, and improves upon it in terms of accuracy and size. It has similar accuracy to AlexNet on ImageNet, with 50x fewer parameters and 510x smaller size. SqueezeNet uses Fire modules as building block for its architecture. A Fire module has one 1x1 “squeeze” convolution layer. It then goes into expand layers, which have 1x1 and 3x3 filters. The squeeze layers decrease input channels seen by the 3x3 filters, resulting in reduced number of parameters. It then adds bypass connection around some Fire modules, which allows the layers to be not fully connected. This bypass connection is similar to the residual connection in ResNet [11].

ShuffleNet [42] is designed for mobile devices with very limited computing powers. On ARM-based devices, it achieves around 13x increased speed over AlexNet while keeping similar level of accuracy. In ShuffleNet, a convolution operates only on its corresponding input channel group. These groups reduce computational cost by a significant margin. And with channel shuffle operation, it becomes possible to avoid stacking multiple group convolutions. These combine into a shufflenet unit. They then add a 3x3 average pooling and substitute the element-wise addition with channel concatenation. This makes enlarging channel dimension easier, and thus allows for more efficient training.

EfficientNets [37] are a family of 8 baseline networks that scale up on MobileNet and ResNet. These networks are uniformly scaled in depth, width, and resolution using a factor termed *compound coefficient*. This method is called *compound scaling*. With this, EfficientNet-~B7 achieves 84.1% top-1 accuracy on ImageNet, with a 8.4x smaller size, and 6.1x faster speed.

MobileNetV2 [35] is another lightweight architecture for mobile and embedded devices. It is made up using *bottleneck* layers. These layers are connected to form an inverted residual structure. It also uses depthwise convolutions to filter sources of non-linearity, which in turn reduces the number of parameters. That is then followed by another linear bottleneck layer to reduce the number of input channels to the next layer.

DenseNet [43] establishes direct connections between each layer and all subsequent layers, enhancing feature reuse. This architecture allows for high efficiency with fewer parameters.

#### 3.2.2 ViT models

**Figure 2:**
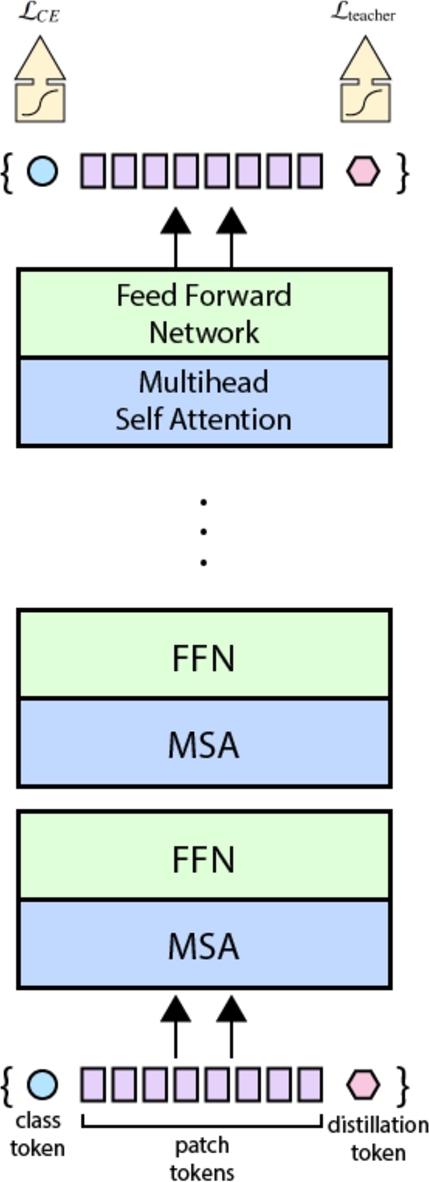
DeiT Architecture.

Data-efficient image transformers (DeiT), like regular vision transformers, rely on neural networks purely based on attention. However, to overcome the need for training the model on a huge dataset (“hundred of millions of images” [41]), it adopts a *teacher-student* strategy. The teacher model (a ConvNet, or a mixture of classifiers) outputs a *soft* label, which the student model (a transformer) tries to match. A hard label is the maximum score of the teacher's output, while a soft label is the softmax function's output vector of the same. This is done through knowledge distillation [12]. This process allows the student model to be trained on a smaller dataset, while still achieving a similar accuracy to the teacher model. The teacher model is discarded after training. It obtains upto 85.2% top-1 accuracy on ImageNet. The DeiT architecture is shown in Fig. 2.

**Figure 3:**
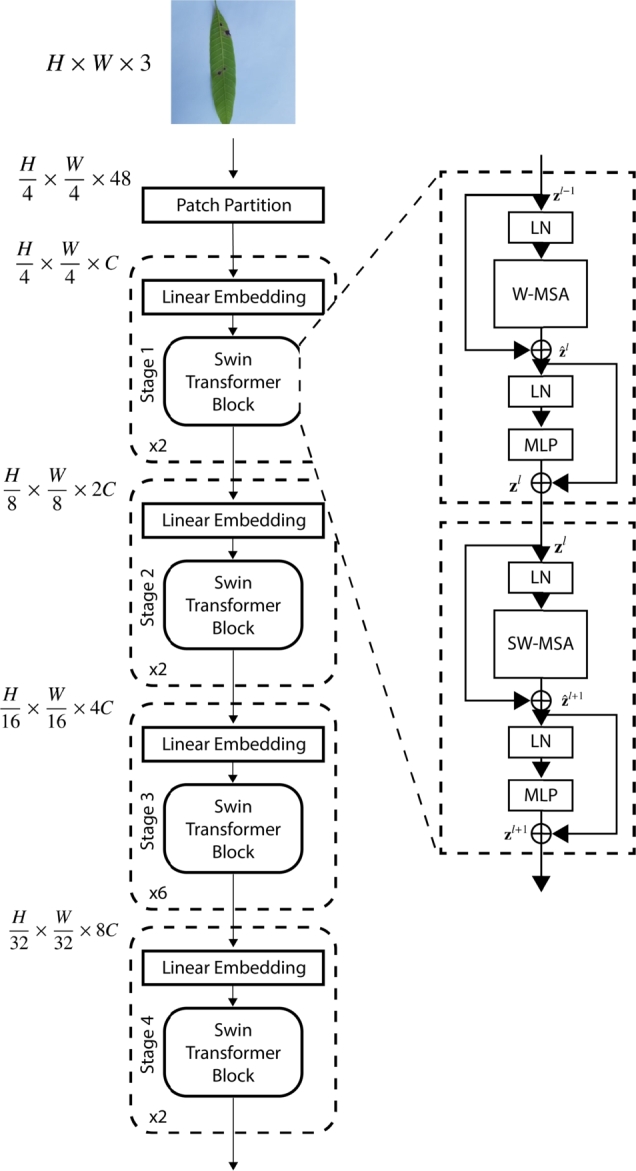
SwinTransformer Architecture.

SwinTransformer [19] adopts a *shifted windowing* scheme, primarily for the issues concerning higher inconsistencies in both large-scale visual entities and images with high pixel density as opposed to words in text. This approach limits self-attention computations within non-overlapping windows while maintaining cross-windows connections. This is illustrated in Fig. 3. It achieves 87.3% accuracy on ImageNet-1K for image classification tasks and 58.7 box AP for object detection problems, the latter surpassing state-of-the-art systems by a large margin. This hierarchical setup has linear complexity with respect to image size, proving beneficial for multilayer perceptron models.

### 3.3 Proposed approach

**Figure 4:**
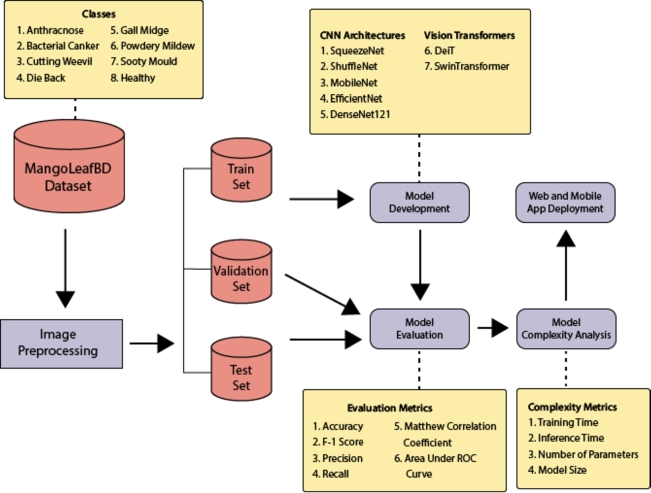
Methodology of the proposed approach.

Our methodology is illustrated in Fig. 4. After collecting the data and splitting them into train, test, and validation sets, we apply a uniform preprocessing step to all the images. Each image is first randomly cropped from the center and then resized to 224 x 224 pixels. We then apply a random horizontal flip to it. After that, we normalized the image to have the default mean and standard deviation of ImageNet. We then trained the models using the configurations in Table 2, Table 3. The number of epochs is the number of times the entire training dataset is processed through the network. The CNN models were trained for 15 epochs and ViT models were trained for 5 epochs. Optimizers are used to update the weights of the models during the training. We used Adam [17] as an optimizer for all models. The loss function is used to measure the error between the predicted and actual labels. We use cross-entropy loss function here. The learning rate is the step size at each iteration while moving toward the minima of the loss function. The batch size is the number of samples that are propagated through the network at each iteration. Each model used pre-trained weights to leverage the benefits of transfer learning. The models were trained on a machine with the specifications presented in Table 4. Each model was evaluated on the test set. We measured the size, training time, and inference time of each model.

**Table 2:** Epochs and Learning rates used to train each model.

Model	Epoch	Learning Rate
SqueezeNet	15	1e-5
ShuffleNet	15	1e-4
MobileNetV2	15	1e-5
EfficientNet	15	2e-5
DenseNet121	15	5e-4
DeiT	5	1e-6
SwinTransformer	5	2e-6

**Table 3:** Hyperparameters configuration.

Hyperparameter	Value
Batch Size	32
Input Size	224, 224, 3
Optimizer	Adam
Loss Function	Categorical Cross-entropy
Activation	Softmax
Patch Size	16,16

**Table 4:** Specification of the machine used for training.

Name	Parameter
Memory	16 GB
Processor	Intel(R) Xeon(R) CPU @ 2.00GHz
GPU	NVIDIA P100 16 GB
OS	Debian GNU/Linux 8

### 3.4 Knowledge distillation

The teacher model in a teacher-student strategy is typically a large and high-capacity model trained on a very large dataset. In the case of DeiT, the current state-of-the-art result in distillation is achieved through pretraining the model on the JFT-300M dataset [36] at resolution 512. The student model, which is the DeiT in this case, is trained to follow the output of the teacher model. Instead of solely relying on ground truth labels, the student model learns from the teacher model's predictions. These predictions also include feature representations of hidden layers. The distillation loss is a combination of the traditional classification loss and a difference between the teacher's and the student's output. In the case of the dataset we used, the teacher-student strategy allows the DeiT model to learn effectively from a limited set of annotated images. The pre-trained teacher model, which has been exposed to a vast amount of diverse data, provides a rich source of information that the DeiT model can leverage. The need for a large annotated dataset is mitigated, making the approach more practical.

### 3.5 ViT optimization

As discussed earlier, vision transformers are larger in size than CNNs. This increased size often leads to better accuracy, but it also requires more computational resources. However, our goal is to deploy the best model on mobile devices and web servers for better accessibility. To achieve this, we optimized the trained ViT models to reduce their size while maintaining similar performance. We used the following methods to achieve this.

PyTorch provides a mode named TorchScript, that allows using the PyTorch JIT compiler. This creates serializable models from existing python code. A TorchScript program can run independently without relying on any python dependencies. This allows us to use the model on any platform without relying on the overhead of python packages.

Quantization is another method that allows us to store the tensors at lower bitwidths than floating point precision. The new quantized model executes some or all operations on tensors with reduced precision. While this sounds weak in theory, it has been shown to have minimal impact on the accuracy. That is because the model is trained on floating point precision, and most models are overparameterized. This gives us significant amount of room for error. A mapping function is used to map the floating point values to integer space. A linear transformation given by Equation (1) is often used as a mapping function.

(1) $$Q(r)=round(\frac{r}{S}+Z)$$

Here, the quantization parameters are *S* (scaling factor) and *Z* (zero-point). The scaling factor is simply the ratio of the input range to the output range, and the zero-point acts as a bias to ensure that a 0 input maps to a 0 output in the quantized space.

### 3.6 Performance measurement

We used accuracy, precision, recall, F1-score, and Matthews Correlation Coefficient (MCC) to measure the performance of each model. The equations for calculating these metrics are shown in Equation (2), (3), (4), (5), and (6).

(2) $$Accuracy=\frac{TP+TN}{TP+TN+FP+FN}$$

(3) $$Precision=\frac{TP}{TP+FP}$$

(4) $$Recall=\frac{TP}{TP+FN}$$

(5) $$\text{F1-score}=2\times \frac{Precision\times Recall}{Precision+Recall}$$

(6) $$\text{MCC}=\frac{TP\times TN-FP\times FN}{\sqrt{\left(TP+FP)(TP+FN)(TN+FP)(TN+FN\right)}}$$

Here, FP, FN, TP, and TN are the number of false positives, false negatives, true positives, and true negatives, respectively.

## 4 Results

### 4.1 Evaluation results

**Figure 5:**
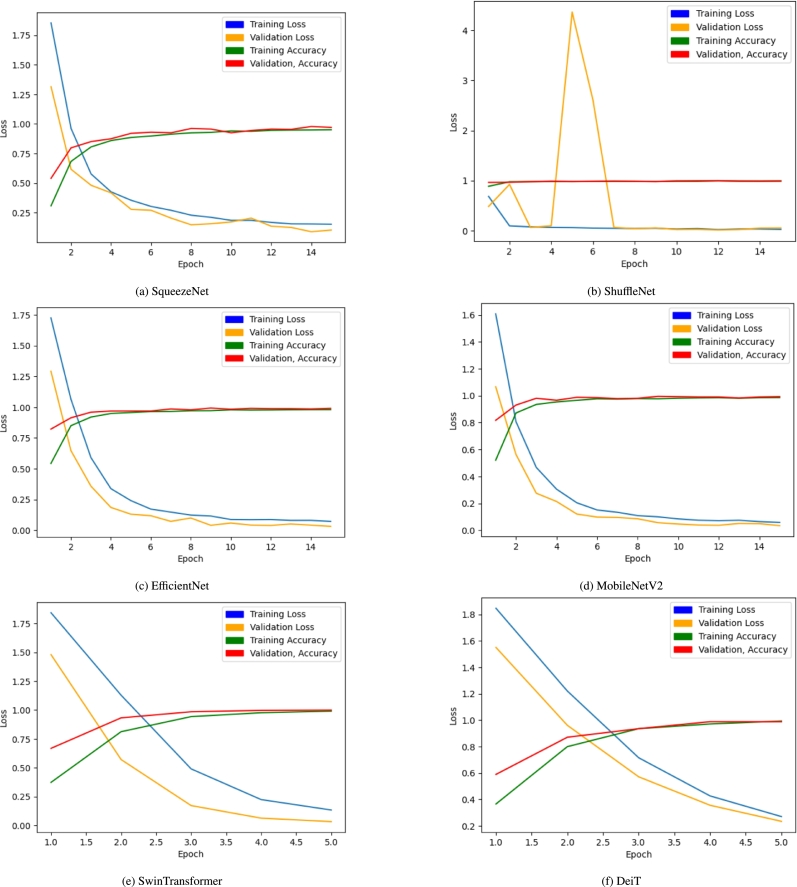
Loss and Accuracy of the trained models.

The loss and accuracy plots for the trained models are presented in Fig. 5a to Fig. 5f. The performance metrics for each model are presented in Table 5. The DeiT model outperforms all other CNN models in every metric despite being trained for only one-third of the epochs. It achieves 99.75% accuracy and 99.749% F-1 score. The SwinTransformer model follows closely, with a similar number of epochs and an accuracy of 99.50%. ShuffleNet performs the best among the CNN models, achieving 99.50% accuracy.

**Table 5:** Performance metrics of the trained models.

Model	EPOCH	ACC	F1	PRE	REC	MCC
SqueezeNet	15	96.50	96.52	96.50	96.54	96.60
ShuffleNet	15	99.50	99.50	98.97	99.54	99.35
MobileNetV2	15	98.79	98.754	98.797	98.750	98.86
EfficientNet	15	98.75	98.81	98.79	98.77	98.75
DenseNet121	15	96.75	96.78	96.75	96.804	96.55
SwinTransformer	5	99.50	99.502	99.519	99.500	99.430
**DeiT**	**5**	**99.75**	**99.749**	**99.754**	**99.750**	**99.72**

**Figure 6:**
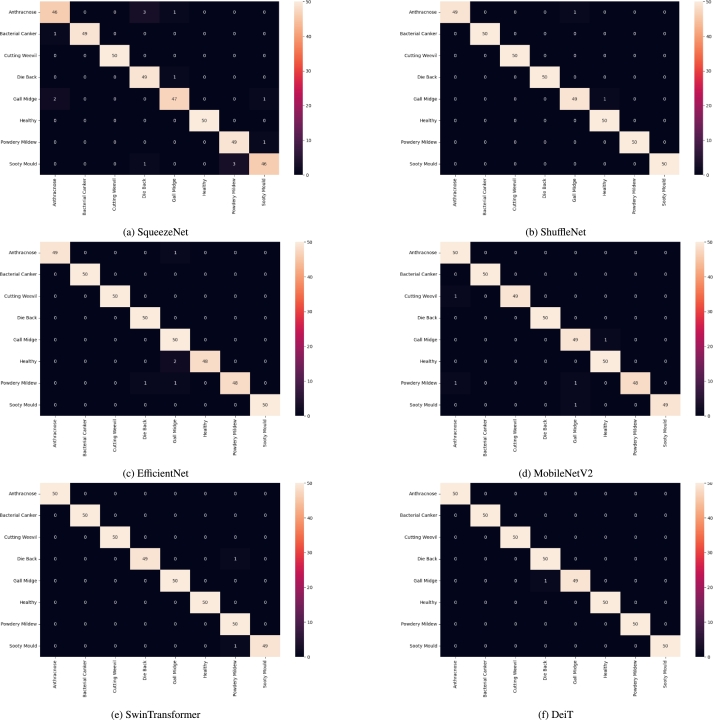
Confusion matrices for all six models.

Fig. 6 shows the confusion matrices for each model tested on the test set. The DeiT model has the highest number of correct predictions, with only one misclassification (Fig. 6f). It predicts one gall midge infected leaf as a member of the die back class. SwinTransformer has two misclassifications (Fig. 6e). ShuffleNet misclassified two images while MobileNet and EfficientNet inaccurately predicted five images each. SqueezeNet once again performed significantly worse, misclassifying four samples of the Anthracnose class, which is evidently the easiest to identify (Fig. 6a-Fig. 6d).

**Figure 7:**
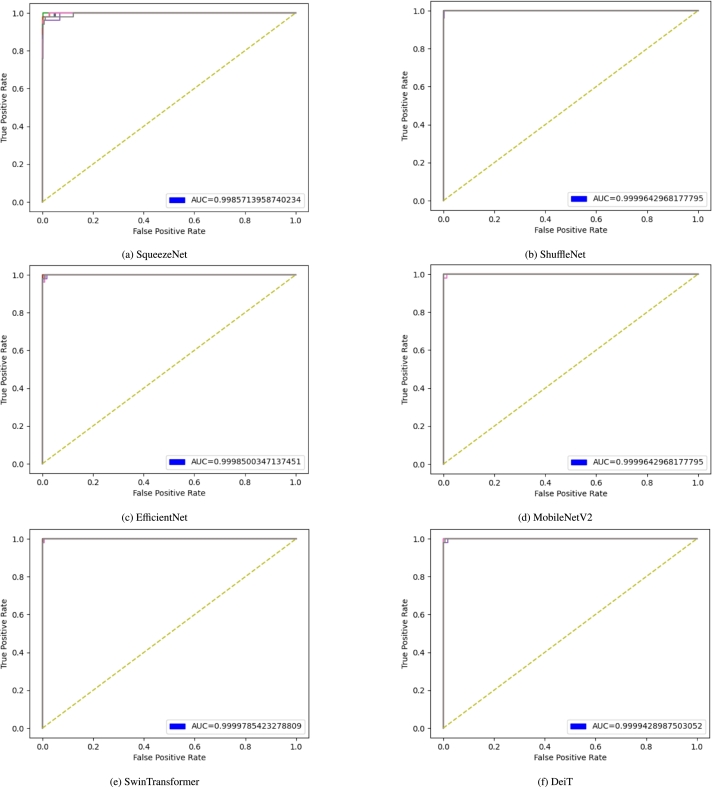
Receiver Operating Characteristic (ROC) curves for all six models.

Fig. 7 illustrates the Receiver Operating Characteristic curve for each model. ROC shows the relationship between sensitivity and specificity, which in turn measures how much the model is capable of distinguishing between classes. The area under the curve (AUC) can be used as a criterion for this measurement. Classifiers that produce curves closer to the top-left corner indicate better performance. While the average AUC for all models are similar, SqueezeNet attains a lower score again (Fig. 7a). ShuffleNet gives the best curve among all CNN models although DeiT and SwinTransformer produce the best curves overall, with high average AUC (Fig. 7a-Fig. 7f).

### 4.2 Visualizing attention maps

**Figure 8:**
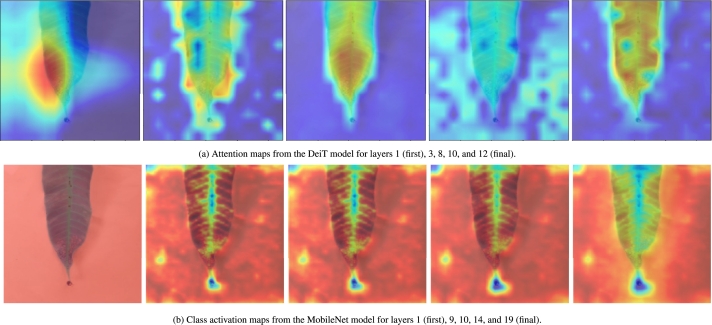
Attention and activation maps for respective models (ordered left to right). Visualization is done with GradCAM.

Fig. 8a visualizes the attention maps of all layers of the DeiT model for a sample image of the Anthracnose class. The maps show us which parts of the image the model focuses on for that class. These are akin to the feature extraction of a CNN model. For reference, Fig. 8b shows the feature maps of the same image for MobileNet. While both networks fixate on meaningful regions, the attention maps are more compact on the symptom regions of the disease.

### 4.3 Model complexity

Table 6 shows the number of parameters, size, inference time, and training time for each model. Additionally, we also test the same for the optimized DeiT model. SqueezeNet has the lowest number of parameters, and is the smallest in size. It also has the lowest inference time. The DeiT model has the highest number of parameters, is the largest in size, and has the highest inference time. The optimized DeiT model has a similar number of parameters to the SqueezeNet, attaining a 91.7% reduction. The size of the model is only 8.5% of the original model, reduced to 88.47 MB. It also achieves a lower inference time. The SwinTransformer, despite having a high number of parameters, completes training in the shortest time. It also has a smaller inference time than DeiT.

**Table 6:** Complexity of the trained models and the optimized DeiT. Inference times were measured on an Intel(R) Xeon(R) CPU at 2.20GHz.

Model	Train time	Parameters	Size	Inference time
SqueezeNet	269.83 s	726.6 k	8.78 MB	20.80 ms
ShuffleNet	323.63 s	5.36 M	64.72 MB	40.76 ms
MobileNetV2	332.26 s	2.23 M	27.19 MB	42.28 ms
EfficientNet	387.06 s	4.01 M	48.7 MB	45.01 ms
DenseNet121	509.27 s	6.96 M	84.47 MB	119.34 ms
SwinTransformer	257.32 s	27.58 M	110.30 MB	206.02 ms
DeiT	321.43 s	85.8 M	1.03 GB	346.87 ms
**Optimized DeiT**	N/A	781 k	88.47 MB	315.78 ms

### 4.4 Comparison with other work

We compare the performance of the optimized DeiT model against four of the most recent work done with the MangoLeafBD dataset. Rizvee et al. [33] develop on top of the AlexNet architecture and present LeafNet, a deep CNN, that achieves 99.5% accuracy. Mahmud et al. [21] fine-tune the DenseNet architecture to reach an accuracy of 99.44% on the MangoLeafBD dataset. Salamai et al. [34] propose a solution with visual modulation blocks. They achieve an accuracy of 99.23%, precision of 99% and an F1-score of 99%. Mahbub et al. [20] present a lightweight CNN architecture that manages to achieve 98% accuracy, 97.62% precision, and 97.50% recall. Table 7 shows the comparison of these results against our proposed optimized DeiT model. The proposed model achieves significant edge over the others in every metric.

**Table 7:** Comparison of results of the proposed model against other work with MangoLeafBD.

Model	Method	Year	Accuracy	Precision	Recall	F1-score
Salamai et al. [34]	Visual Modulation Networks	2023	99.23	99.01	99.03	99.02
Mahmud et al. [21]	DenseNet	2024	99.44	97.12	97.50	97.62
Rizvee et al. [33]	Deep CNN	2024	99.55	99.50	97.45	97.47
Mahbub et al. [20]	Lightweight ConvNet	2023	98.00	97.62	97.50	97.50
**Optimized DeiT (Proposed)**	Vision Transformers	2024	**99.75**	**99.754**	**99.75**	**99.749**

We also present the performance of our model on the Harumanis Mango Leaves dataset [14] and the Plant Disease Fruits dataset [30], as shown in Table 8. It is observed that the model achieved very high accuracy on both datasets.

**Table 8:** Evaluation results of the proposed model against other works of predicting mango leaf diseases.

Model	Dataset	No. of classes	Accuracy	Precision	Recall	F1-score
Salamai et al. [34]	Harumanis Mango Leaves [14]	3	96.77	96.34	96.27	96.30
Optimized DeiT (Proposed)	Harumanis Mango Leaves [14]	3	97.16	97.02	97.10	97.15
Optimized DeiT (Proposed)	Plant Disease Fruits^a^[30]	2	96.11	96.01	96.30	96.11

a For the Plant Disease Fruits dataset, only the images of healthy and diseased mangoes were used.

### 4.5 Server deployment and inference

**Figure 9:**
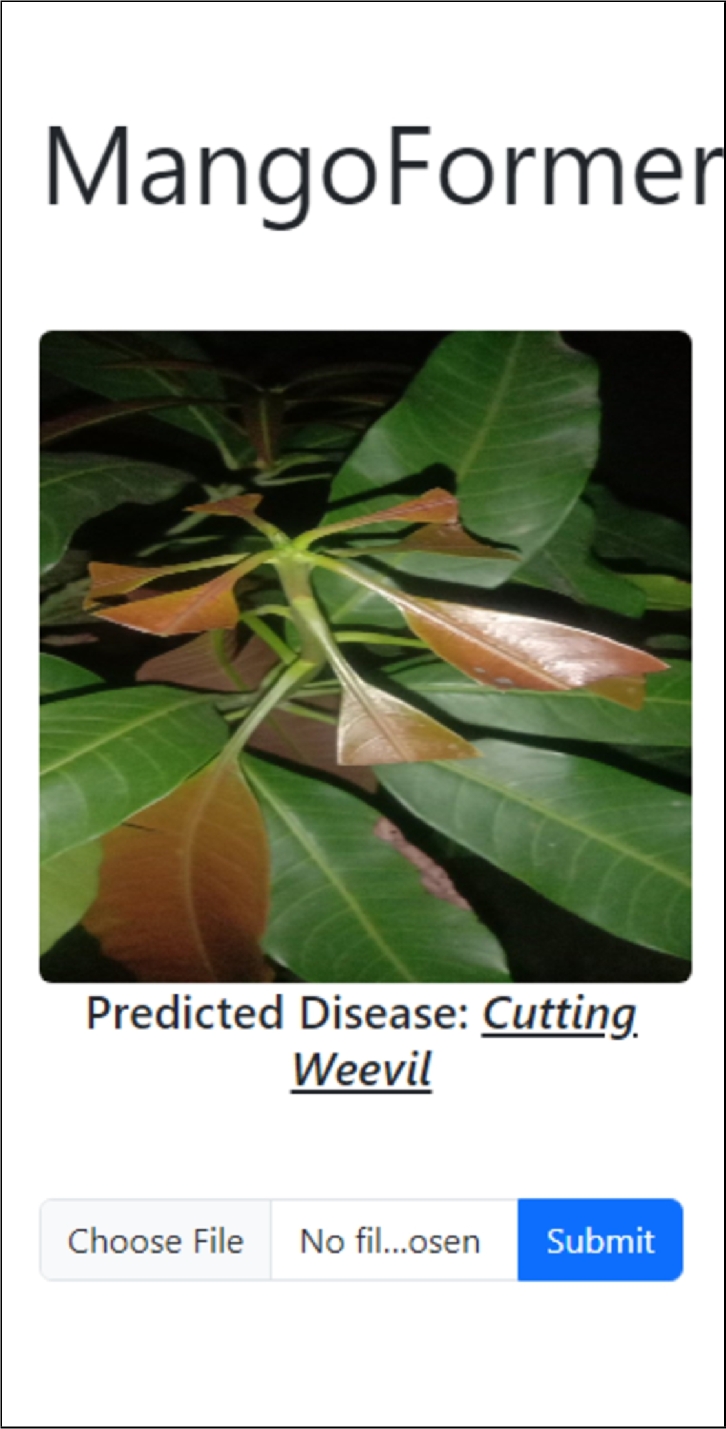
MangoFormer mobile app. Image captured from a local mango tree in Gazipur.

We deployed the models on a server for inference through an API. The API uses a Flask backend written in Python and deployed remotely. The server uses 512 MB of RAM and a shared single-core CPU. All the models took a similar time for inference. The optimized DeiT model was used for the final production build, as it had a smaller size. Using the API, we also developed a mobile app that can capture a photo and identify if it contains a diseased mango leaf, as shown in Fig. 9.

## 5 Discussion

The results in Section 4 show that SqueezeNet is the smallest CNN in size with the least number of parameters. But it comes with the disadvantage of having a significantly lower accuracy. ShuffleNet, on the other hand, achieves a much better accuracy, but it has a larger size and more parameters.

On the other hand, the ViT models prove useful in both aspects. Both models achieve better accuracies than all CNN models. Despite its large size, the DeiT model takes a similar amount of time to train without compromising accuracy. This results in a lower computational cost and less resource usage.

For our case, the optimized DeiT model proves to be the most beneficial by being both fast and accurate. It has a competitive size too, which allows us to deploy it to a server. The model also has a short inference time, making it convenient for real-time use.

The study is not without its limitations. The dataset is a small one. Using a more diverse dataset in terms of origin, disease type, and environmental conditions would be more representative of real world scenarios. Also, a server deployment forces the models to run only on CPUs. Being able to run the deployments on GPUs would allow us to use much larger and complex models without sacrificing inference time.

Our solution still outperforms state-of-the-art CNN models. This shows that ViTs are a viable alternative to CNNs. The transformer architecture is also more flexible to fine-tuning [22]. This means that vision transformers can scale better than CNNs with a more comprehensive dataset. This is a promising result for the future of vision transformers.

## 6 Conclusion

In this study, we discussed how mango leaves are affected by different diseases. We used a dataset of seven different mango diseases. We compared the performance of popular deployable CNNs to vision transformer architectures in identifying the diseases. We then optimized the vision transformer models, with already the highest accuracy, to have a size suitable for deployment on server and mobile devices. Users can use the mobile app for real-time diagnosis of mango leaves directly from the field. We hope that this work will help farmers for early disease identification and prevention. We also hope that this approach will be adopted for other image classification problems and will serve as a foundation for further research in this domain.

## Funding

This research did not receive funding from public, private, or any other non-profit organizations.

## CRediT authorship contribution statement

**Md. Arban Hossain:** Writing – original draft, Methodology, Investigation, Formal analysis. **Saadman Sakib:** Investigation. **Hasan Muhammad Abdullah:** Writing – review & editing, Investigation, Formal analysis. **Shifat E. Arman:** Writing – original draft, Supervision, Project administration, Methodology.

## Declaration of Competing Interest

The authors declare that they have no known competing financial interests or personal relationships that could have appeared to influence the work reported in this paper.

## Data Availability

The MangoLeafBD dataset [1] used in this study is publicly available on https://data.mendeley.com/datasets/hxsnvwty3r/1. The Harumanis mango leaves dataset [14] is publicly available at https://www.kaggle.com/datasets/mypapit/harumanis-leaves-basic-2021. The Plant Disease Fruits dataset [30] is publicly available at https://www.kaggle.com/datasets/kushless13/plant-disease-fruits.
